# Prognostic factors for soft tissue sarcoma patients with lung metastases only who are receiving first‐line chemotherapy: An exploratory, retrospective analysis of the European Organization for Research and Treatment of Cancer‐Soft Tissue and Bone Sarcoma Group (EORTC‐STBSG)

**DOI:** 10.1002/ijc.31286

**Published:** 2018-02-14

**Authors:** Lars H. Lindner, Saskia Litière, Stefan Sleijfer, Charlotte Benson, Antoine Italiano, Bernd Kasper, Christina Messiou, Hans Gelderblom, Eva Wardelmann, Axel Le Cesne, Jean‐Yves Blay, Sandrine Marreaud, Nadia Hindi, Ingrid M.E. Desar, Alessandro Gronchi, Winette T.A. van der Graaf

**Affiliations:** ^1^ Klinikum der Universität München, Interdisciplinary Tumor Center (CCC LMU), Sarcoma Unit (SarKUM), Marchioninistr. 15 München D‐81377 Germany; ^2^ European Organisation for Research and Treatment of Cancer (EORTC) Data Centre, Avenue Mounier 83/11 Brussels B‐1200 Belgium; ^3^ Erasmus MC Cancer Institute, Groene Hilledijk 301 Rotterdam EA 3075 The Netherlands; ^4^ The Sarcoma Unit Royal Marsden NHS Foundation Trust, Fulham Road London SW3 6JJ United Kingdom; ^5^ Department of Medical Oncology, 229 cours de l'Argonne Institut Bergonie Bordeaux Cedex 33076 France; ^6^ University of Heidelberg, Mannheim University Medical Center, Interdisciplinary Tumor Center, Sarcoma Unit, Theodor‐Kutzer‐Ufer 1‐3 Mannheim D‐68167 Germany; ^7^ Department of Clinical Oncology Leiden University Medical Centre, Albinusdreef 2 Leiden ZA 2333 The Netherlands; ^8^ Gerhard‐Domagk‐Institut für Pathologie, Universitätsklinikum Münster Münster 48149 Germany; ^9^ Institut Gustave Roussy Villejuif France; ^10^ Department of Medicine, NetSARC and LYRIC Centre Leon Berard Lyon France; ^11^ Department of Medical Oncology University Hospital Virgen del Rocio and Biomedicine Research Institute (IBIS) Sevilla Spain; ^12^ Radboud University Medical Center Nijmegen the Netherlands; ^13^ Department of Surgery‐Sarcoma Service Fondazione IRCCS Istituto Nazionale dei Tumori, Via Venezian 1 Milan 20133 Italy; ^14^ Division of Clinical Studies Institute of Cancer Research Sutton United Kingdom

**Keywords:** soft tissue sarcoma, prognostic factors, lung metastases, EORTC

## Abstract

The prognosis of adult soft tissue sarcoma (STS) patients with metastases is generally poor. As little is known about the impact of the involvement of different metastatic sites and the extent of pulmonary lesions on the outcome for patients receiving first‐line chemotherapy, we aimed to establish prognostic factors for STS patients with lung metastases only. A retrospective, exploratory analysis was performed on 2,913 metastatic STS patients who received first‐line chemotherapy. Detailed information from 580 patients who had lung metastases only, was used for prognostic factor analysis. Patients with lung metastases only were more often asymptomatic and had undergone complete primary tumor resection more frequently compared to patients with additional metastases outside the lung or without lung metastases. For extremity STS, the incidence of lung metastases only was much higher compared to non‐extremity STS. Lung involvement only was an independent favorable prognostic factor for overall survival (OS) with regard to metastatic site. Within this subgroup, in a multivariate model, other factors associated with improved OS included: good performance status (PS), no progression at primary site, low histological grade, younger age, long interval between initial diagnosis and trial registration, and smaller diameter of the largest lung lesion. This unique analysis on prognostic factors in STS patients with lung metastases confirms well‐known patient factors (such as age and PS), and tumor characteristics (including tumor grade, interval between primary diagnosis, and metastases), but also identifies diameter of the largest lung lesion as a new prognostic factor. Knowledge about these factors may support decision‐making within multidisciplinary tumor boards.

## Abstract 2

**What's new?**

About 25% of soft tissue sarcoma patients develop lung metastases but prognostic factors for those who cannot get operated remain scarce. Here the authors performed a retrospective study of datasets obtained from 580 patients where metastases have not spread beyond the lung and who received first‐line chemotherapy but no resection. Interestingly, the largest diameter of a lung lesion emerged as a new prognostic factor, a finding, which may influence clinical decisions in the future.

## Highlights

• We established prognostic factors for soft tissue sarcoma patients with lung metastases only on the largest dataset of 580 patients.
• The largest diameter of a lung lesion has been proven among others for the first time to serve as a new prognostic factor.
• Knowledge about these factors may support decision‐making within multidisciplinary tumor boards.

## Introduction

Soft tissue and visceral sarcomas represent a rare group of mesenchymal tumors with greater than 70 different subtypes. The incidence in Europe and the United States is less than 5 per 100.000 per year.^1^, ^2^ They occur at various anatomic sites with extremities (43%), trunk (10%), visceral (19%), retroperitoneum (15%), and head and neck (9%) as the most common primary sites.^3^ The three stage grading system established by the French sarcoma group (FNCLCC) discriminates rarely metastasizing grade 1 tumors from grade 2 and 3 tumors with high metastatic potential. Other important characteristics leading to an increased risk of metastasis formation are size and depth of the primary tumor.^4^ Most soft tissue sarcoma (STS) metastasize hematogenously with the lung as main target organ.^5^ Exceptions are synovial sarcoma, clear cell sarcoma, rhabdomyosarcoma, and epithelioid sarcoma, which also spread via the lymphatic system.^6^

Based on previous analyses, approximately 20–25% of all STS patients will develop pulmonary metastases (40–60% for high grade tumors), which will become clinically evident usually in the first 2 years following diagnosis.^7^ For extremity STS, radiotherapy in addition to surgical removal of the tumor has been shown to reduce the risk of local recurrence but it has no impact on survival.^8^, ^9^ Systemic spread seems to be an early event in STS. Amputation of extremity tumors compared to limb sparing procedures did not result in improved survival rates.^10^ Surgical resection of metachronous lung metastases is always considered in the case of limited disease and longer interval since primary surgery as it has been associated with long‐term survival.^11^, ^12^

Prognostic factors associated with improved survival after pulmonary metastasectomy are based on a variety of surgical series. Factors associated with improved survival are a limited number of metastases (≤3), long disease‐free interval (>12 months), and the ability to completely resect the pulmonary lesions.^13^, ^14^ In contrast, it was recently shown that progression on preoperative chemotherapy before pulmonary metastasectomy was an independent negative prognostic factor for survival.^15^

In metastatic patients not amenable to surgery, palliative chemotherapy is the treatment of choice. Median overall survival for these patients is about 12 months, but appears to be rising in certain subtypes such as leiomyosarcomas with the arrival of new agents.^16^ Doxorubicin is the most active drug for metastatic STS and remains the standard first‐line therapy for these patients. The most recent randomized phase III trial comparing doxorubicin with doxorubicin plus ifosfamide did not show an overall survival benefit for the combination therapy.^17^ Furthermore, the approved second‐line agent trabectedin did not improve progression free survival and had more toxicity compared to doxorubicin, and similar results were observed comparing the combination of gemcitabine and docetaxel with doxorubicin in the first‐line therapy.^18^, ^19^

Recently, olaratumab, an anti‐PDGFRα monoclonal antibody, received conditional approval based on overall survival benefit in combination with doxorubicin over doxorubicin alone based on data from a randomized phase II study. Results of the phase 3 study are awaited with great interest.^20^

The aim of the present retrospective exploratory analysis, on data from the EORTC database on 2,913 metastatic STS patients who received first‐line chemotherapy, was to identify prognostic and predictive factors for overall survival (OS) and progression‐free survival (PFS) of 580 patients with lung metastases only.

## Patients and Methods

### Patients

In total, 3,708 patients were treated in fifteen EORTC advanced soft tissue sarcomas trials. We excluded patients without metastases who received prior adjuvant or palliative chemotherapy and who were diagnosed with a gastrointestinal stromal tumor (GIST), limiting the current analysis to 2,913 patients. For the prognostic factor analysis, 1,078 patients with lung metastases only were selected. Detailed information on lung metastases (such as the number and diameter of lesions) had been collected in 10 of the 15 first‐line studies (62,883, 62,901, 62,903, 62,912, 62,941, 62,953, 62,971, 62,012, 62,061, and 62,091), which finally led to data of 580 patients that qualified for the prognostic factor analysis.

### End‐points

The end points of this analysis were PFS and OS. PFS is defined as the time interval between the date of randomization or the date of prospective registration in the nonrandomized trials and the date of first report of progression or death, whichever comes first. The patients who are alive and without progressive disease at the last follow up date are censored. Likewise, OS is computed from the date of randomization in the randomized trials or the date of prospective registration in the nonrandomized trials until the date of death. Patients who are alive at the last follow up date are censored.

### Explored covariates

The variables included in the study were the demographic data, the previous history of the sarcoma, the treatment, and the histology. For the prognostic factor analysis, the characteristics of the pulmonary lesions in particular the diameter of the largest lesions and the total number of lesions were investigated. The demographic variables included age (by 10 years), sex, and performance status before the start of chemotherapy. Performance status (PS) was measured on the WHO scale (except for two trials in which it was retrospectively converted from Karnofsky scale to the WHO scale). As few patients had a level 3 PS, the level 2 and 3 were combined in the same category named “PS 2+.” Variables related to the history of sarcoma included prior radiotherapy as well as the time since the first diagnosis of sarcoma (in years). Prior chemotherapy was an exclusion criterion. For the histological diagnosis and the grade, we used the centrally reviewed diagnosis when available (around 60%), otherwise the local diagnosis was used. Histological subtypes were aggregated into the four most common groups: leiomyosarcoma, synovial sarcoma, liposarcoma, and others. The treatment was aggregated in the four categories: *anthracyclines alone* (doxorubicin 1 ∗ 75 mg/m^2^, caelyx 1 ∗ 50 mg/m^2^, epirubicin 1 ∗ 75 mg/m^2^, epirubicin 3 ∗ 50 mg/m^2^, epirubicin 1 ∗ 150 mg/m^2^), *ifosfamide alone* (ifosfamide 5 g/m^2^/24 hr continuous infusion, ifosfamide 3 ∗ 3 g/m^2^, ifosfamide 9 g/m^2^/72 hr continuous infusion, ifosfamide 12 g/m^2^/72 hr continuous infusion), the *combination of doxorubicin and ifosfamide* (doxorubicin 50 mg/m^2^ + ifosfamide 5 g/m^2^/24 hr continuous infusion, doxorubicin 75 mg/m^2^ + ifosfamide 5 g/m^2^/24 hr continuous infusion), CYVADIC (cyclophosphamide 500 mg/m^2^, vincristine 1.5 mg/m^2^, doxorubicin 50 mg/m^2^, dacarbazine 750 mg/m^2^), and *other* (brostallicin 10 mg/m^2^, trabectedin 1.5 mg/m^2^/24 hr or 1.3 mg/m^2^/3 hr) (Supporting Information Appendix Table 1). This variable was used as a stratification factor in the univariate analyses.

### Statistical methods

All baseline variables were described. The categorical data were summarized by the frequencies and percentages, and the continuous covariates were summarized with median, range and numbers of non‐missing observations. The statistical test used for comparison was a χ^2^ (or a fisher) test for categorical covariates. A Kruskall–Wallis test was used for covariates with more than two ordered categories or for continuous variables. Median follow‐up was calculated from reverse Kaplan–Meier estimates. PFS and OS were estimated using the Kaplan–Meier estimate and compared between subgroups of metastatic involvement using a Cox proportional hazards models stratified by treatment and study to try to account for potential heterogeneity among the trials. The potential prognostic value of all factors was first investigated by univariate analyses, using a univariate Cox model stratified by treatment. The overall and progression free survival curves were presented for factors that were significant. Factors included in the multivariate model (also stratified by treatment) were identified by a backward selection procedure which included all the covariates in the model and removed, one at a time, those whose *p* values was higher than 0.05. Multivariate analysis required complete information for all patients on all covariates included in the model. To protect against a considerable loss of information for the multivariate analysis in case of substantial missing data in one or more of the covariates, we considered for some covariates the “missing” as a separate category in these models (prior surgery, primary site involved, site of primary and grade). The statistical significance was set at 0.05 for all the analyses in this report.

## Results

Baseline characteristics of all 2,913 metastatic STS patients were categorized according to the site of metastatic involvement “lung lesions only,” “other lesions only,” and “both type of lesions” (Table 1). In the univariate analysis, the patients’ characteristics differed across the populations for treatment, gender, PS, age, time between initial diagnosis, and registration, prior surgery, prior radiotherapy, histology, histopathological grade, site of primary tumor, and whether the primary site was involved.

**Table 1:** Characteristics of 2,913 metastatic sarcoma patients receiving first‐line chemotherapy\

	Lesions			Total (*N* = 2,913)	
	Lung lesions only (*N* = 1,078)	Other lesions only (*N* = 936)	Both (*N* = 899)		*p*‐value
	*N* (%)	*N* (%)	*N* (%)	*N* (%)	
**Treatment**					<0.001^a^
** Anthracyclines**	383 (35.5)	388 (41.5)	347 (38.6)	1118 (38.4)	
** DOX + IFO**	354 (32.8)	300 (32.1)	294 (32.7)	948 (32.5)	
** CYVADIC**	157 (14.6)	109 (11.6)	89 (9.9)	355 (12.2)	
** Ifo ALONE**	138 (12.8)	99 (10.6)	98 (10.9)	335 (11.5)	
** Other**	46 (4.3)	40 (4.3)	71 (7.9)	157 (5.4)	
**Gender**					0.007^a^
** Male**	576 (53.4)	470 (50.2)	416 (46.3)	1462 (50.2)	
** Female**	502 (46.6)	466 (49.8)	483 (53.7)	1451 (49.8)	
**Performance status**					<0.001^a^
** PS 0**	548 (50.8)	394 (42.1)	353 (39.3)	1295 (44.5)	
** PS 1**	445 (41.3)	453 (48.4)	450 (50.1)	1348 (46.3)	
** PS 2+**	71 (6.6)	79 (8.4)	84 (9.3)	234 (8.0)	
***Missing***	*14 (1.3)*	*10 (1.1)*	*12 (1.3)*	*36 (1.2)*	
**Age**					
** ≤40 years**	292 (27.1)	187 (20.0)	192 (21.4)	671 (23.0)	
** 40–50 years**	231 (21.4)	200 (21.4)	225 (25.0)	656 (22.5)	
** 50–60 years**	303 (28.1)	291 (31.1)	268 (29.8)	862 (29.6)	
** >60 years**	234 (21.7)	252 (26.9)	204 (22.7)	690 (23.7)	
***Missing***	*18 (1.7)*	*6 (0.6)*	*10 (1.1)*	*34 (1.2)*	
** Median**	50	52	51	52	<0.001^b^
** Range**	16–88	10–80	17–84	10–88	
** Q1–Q3**	39–59	43–61	42–60	41–60	
**Time between initial diagnosis and registration**					
** ≤6 months**	456 (42.3)	488 (52.1)	436 (48.5)	1380 (47.4)	
** 6–12 months**	159 (14.7)	91 (9.7)	100 (11.1)	350 (12.0)	
** 1–2 years**	195 (18.1)	102 (10.9)	123 (13.7)	420 (14.4)	
** >2years**	224 (20.8)	192 (20.5)	193 (21.5)	609 (20.9)	
***Missing***	*44 (4.1)*	*63 (6.7)*	*47 (5.2)*	*154 (5.3)*	
** Median (months)**	9	6	7	7	0.001^b^
** Range (months)**	0–312	0–347	0–250	0–347	
** Q1–Q3 (months)**	2–21	1–21	2–22	2–21	
**Prior surgery**					<0.001^a^
** No surgery**	90 (8.3)	91 (9.7)	64 (7.1)	245 (8.4)	
** Non‐optimal surgery**	136 (12.6)	145 (15.5)	88 (9.8)	369 (12.7)	
** Complete surgery**	437 (40.5)	187 (20.0)	206 (22.9)	830 (28.5)	
** Other/Unknown**	415 (38.5)	513 (54.8)	541 (60.2)	1469 (50.4)	
**Prior radiotherapy**					<0.001^a^
** No**	595 (55.2)	732 (78.2)	511 (56.8)	1838 (63.1)	
** Yes**	416 (38.6)	143 (15.3)	286 (31.8)	845 (29.0)	
***Missing***	*67 (6.2)*	*61 (6.5)*	*102 (11.3)*	*230 (7.9)*	
**Histology**					<0.001^a^
** Leiomyosarcoma**	261 (24.2)	378 (40.4)	281 (31.3)	920 (31.6)	
** Synovial sarcoma**	176 (16.3)	29 (3.1)	75 (8.3)	280 (9.6)	
** Liposarcoma**	67 (6.2)	135 (14.4)	62 (6.9)	264 (9.1)	
** Other**	539 (50.0)	353 (37.7)	459 (51.1)	1351 (46.4)	
***Missing***	*35 (3.2)*	*41 (4.4)*	*22 (2.4)*	*98 (3.4)*	
**Histopathological grade**					<0.001^a^
** Grade 1**	65 (6.0)	115 (12.3)	54 (6.0)	234 (8.0)	
** Grade 2**	261 (24.2)	266 (28.4)	267 (29.7)	794 (27.3)	
** Grade 3**	412 (38.2)	275 (29.4)	325 (36.2)	1012 (34.7)	
***Missing***	*340 (31.5)*	*280 (29.9)*	*253 (28.1)*	*873 (30.0)*	
**Site of primary tumor**					<0.001^a^
** Other than extremity**	349 (32.4)	520 (55.6)	393 (43.7)	1262 (43.3)	
** Extremity**	416 (38.6)	131 (14.0)	294 (32.7)	841 (28.9)	
***Missing***	*313 (29.0)*	*285 (30.4)*	*212 (23.6)*	*810 (27.8)*	
**Primary site involved**					<0.001^a^
** No**	619 (57.4)	329 (35.1)	436 (48.5)	1384 (47.5)	
** Yes**	366 (34.0)	400 (42.7)	333 (37.0)	1099 (37.7)	
***Missing***	*93 (8.6)*	*207 (22.1)*	*130 (14.5)*	*430 (14.8)*	
**Involved sites**					
** Lung lesions only**	1078 (100.0)	0 (0.0)	0 (0.0)	1078 (37.0)	
** Liver lesions**	0 (0.0)	195 (20.8)	86 (9.6)	281 (9.6)	
** Bone lesions**	0 (0.0)	43 (4.6)	107 (11.9)	150 (5.1)	
** Other lesions**	0 (0.0)	491 (52.5)	481 (53.5)	972 (33.4)	
** Liver and bone lesions**	0 (0.0)	8 (0.9)	31 (3.4)	39 (1.3)	
** Liver and other lesions**	0 (0.0)	147 (15.7)	75 (8.3)	222 (7.6)	
** Bone and other lesions**	0 (0.0)	34 (3.6)	85 (9.5)	119 (4.1)	
** Liver, bone, and other lesions**	0 (0.0)	18 (1.9)	34 (3.8)	52 (1.8)	

a χ^2^.

b Kruskall–Wallis.

There were slightly more male patients with lung lesions only, whereas more female patients had metastatic sites including lung and other lesions. This was probably related to the gynecologic sarcomas (*N* = 308) which were associated in 62% of patients with lesions also outside the lungs (Supporting Information Appendix Table 3). A PS of 0 was observed more frequently in the “only lung metastases” group as compared to the other two groups. The median age in the three groups was comparable. The interval between initial diagnosis and registration was the longest for patients with lung metastases only. The majority of patients had a prior surgery of their primary tumor which was more often a complete resection for patients with lung metastasis as compared to the others. About 10% of the patients had synovial sarcoma and liposarcoma (9.6 and 9.1%, respectively), 31.6% had leiomyosarcoma, but the majority had another type of sarcoma (46.4%). Remarkably, synovial sarcoma metastases almost always affected the lungs with only 10% of patients having lesions solely outside the lungs, whereas liposarcoma metastases were mainly located outside the lungs (51.1% other lesions only and 23.5% both types of lesions). The next histologic subtype associated with a high prevalence of metastases sparing the lungs was leiomyosarcoma, with 41.1% of patients showing metastases only outside the lungs. The group of other STS was a mixed group of histologies, whereas nowadays the diagnosis can be better defined than in the earlier studies. Thirty percent of grading data were missing, almost half of the STS had grade 3 and 11% had grade 1. Patients with metastatic lesions only outside the lungs had twice as often a grade I tumor as compared to the other two groups. Information about the localization of the primary tumor was incompletely reported but the majority were non‐extremity STS. These patients developed metastases outside the lungs more frequently as compared to the extremity Group (41.2 vs. 15.6%, respectively) (Table 1).

**Figure 1:**
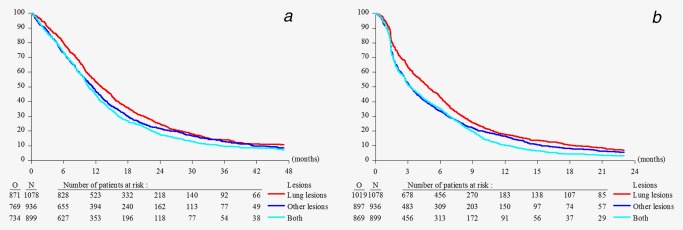
Overall survival (*a*) and progression free survival (*b*) of 2,913 metastatic sarcoma patients receiving first‐line chemotherapy with lung lesions only, other lesions only, or both types of lesions. [Color figure can be viewed at wileyonlinelibrary.com]

At the time of the respective study clinical data cut‐offs, 18.5% of patients were still alive with a median follow‐up time of 16 months. These patients were considered as censored for OS and PFS analysis. In terms of OS patients with lung metastases only had a better prognosis than patients with other metastases only (stratified HR = 1.14; 95% CI, 1.03–1.26) or patients with both types of metastases (stratified HR = 1.24; 95% CI, 1.16–1.43). This was also the case for PFS where patients with lung metastases only had a longer PFS as compared to the group with other lesions only (stratified HR = 1.20; 95% CI, 1.09–1.31) or the group with both type of lesions (stratified HR = 1.31; 95% CI, 1.19–1.44) (Fig. 1).

**Figure 2:**
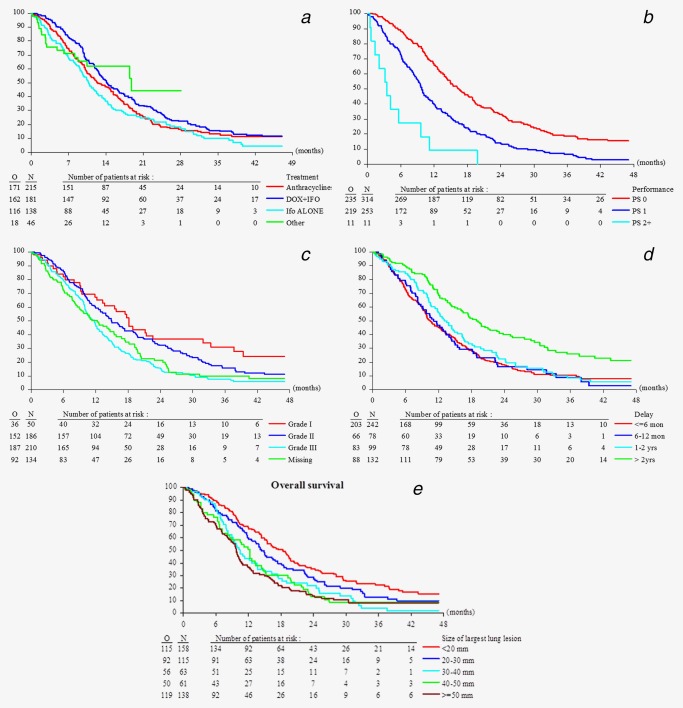
Overall survival in 580 sarcoma patients with lung metastases only by type of chemotherapy treatment (*a*), performance status (*b*), histopathological grading (*c*), time between the initial diagnosis and start of treatment (*d*), and by diameter of the largest lung lesion (*e*). [Color figure can be viewed at wileyonlinelibrary.com]

**Figure 3:**
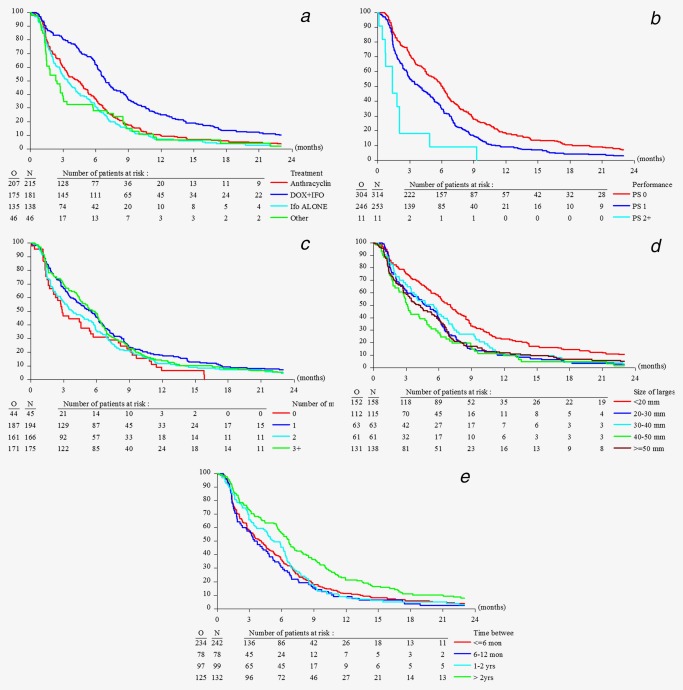
Progression free survival in 580 sarcoma patients with lung lesions only by type of chemotherapy treatment (*a*), performance status (*b*), number of lung lesions (*c*), diameter of the largest lung lesion (*d*), and time between the initial diagnosis and start of treatment (*e*). [Color figure can be viewed at wileyonlinelibrary.com]

### Prognostic factors for OS in patients with measured lung metastases only

In univariate analysis, PS, histopathological grade, time between the initial diagnosis and registration, and the diameter of the largest lung lesion were all significant prognostic factors at the level of 0.05. Choice of chemotherapy regimen had significant impact on OS with a better outcome for doxorubicin plus ifosfamide as compared to doxorubicin alone (Supporting Information Appendix Table 4). Multivariate analysis was performed on a subset of 504 patients with all necessary information available. Performance status was a very strong prognostic factor together with histopathological grade, involvement of primary site, time between the diagnosis and registration, and the diameter of the largest lung lesion (Table 2). We also performed the multivariate analysis adjusting for treatment as a covariate but stratifying by study to account for between study heterogeneity. This resulted in the same final model, with similar HR estimates (data not shown).

**Table 2:** Multivariate analysis, stratified by treatment, of 580 patients (504 with complete covariate information) with lung metastases only for overall survival

Parameter	Levels	Hazard ratio (95% CI)	*p*‐value
Performance status	PS 0	1.00	<0.001 (df = 2)
	PS 1	1.72 (1.39, 2.13)	
	PS 2+	5.17 (2.64, 10.1)	
Primary site involved	No	1.00	0.030 (df = 2)
	Yes	1.39 (1.08, 1.77)	
	Missing	1.26 (0.89, 1.80)	
Histopathological grade	Grade I	1.00	0.011 (df = 3)
	Grade II	1.27 (0.84, 1.90)	
	Grade III	1.72 (1.16, 2.57)	
	Missing	1.39 (0.89, 2.16)	
Time between initial diagnosis	≤6 months	1.00	<0.001 (df = 3)
	1–2 years	1.06 (0.79, 1.41)	
	6–12 months	1.18 (0.87, 1.61)	
	>2 years	0.57 (0.43, 0.75)	
Size of largest lung lesion	<20 mm	1.00	<0.001 (df = 4)
	20–30 mm	1.23 (0.92, 1.65)	
	30–40 mm	2.17 (1.53, 3.08)	
	40–50 mm	1.30 (0.90, 1.89)	
	≥50 mm	2.05 (1.54, 2.73)	

### Prognostic factor for PFS in patients with measured lung metastases only

In univariate analysis, treatment, PS, number of lung lesions, interval between initial diagnosis and registration, and size of the largest pulmonary lesion were significant (Supporting Information Appendix Table 5). Median PFS was highest for patients treated with doxorubicin plus ifosfamide followed by anthracycline monotherapy, ifosfamide and trabectedin or brostallicin. In the multivariate analysis, stratified by treatment, on a subset of 504 patients where all covariates were available, PS, time between the diagnosis and registration and the diameter of the largest lung lesions were found to be significant prognostic factors for PFS (Table 3). The sensitivity analysis adjusting for treatment as a covariate, but stratifying by study, resulted in the same final model, with similar HR estimates (data not shown).

**Table 3:** Multivariate analysis, stratified by treatment, of 580 patients (504 with complete covariate information) with lung metastases only for progression free survival

Parameter	Levels	Hazard ratio (95% CI)	*p*‐value
Performance status	PS 0	1.00	<0.001 (df = 2)
	PS 1	1.29 (1.06, 1.56)	
	PS 2+	3.44 (1.80, 6.55)	
Time between initial diagnosis	≤6 months	1.00	<0.001 (df = 3)
	1–2 years	0.93 (0.72, 1.20)	
	6–12 months	1.21 (0.92, 1.58)	
	>2 years	0.64 (0.50, 0.81)	
Size of largest lung lesion	<20 mm	1.00	0.024 (df = 4)
	20–30 mm	1.30 (1.00, 1.69)	
	30–40 mm	1.55 (1.14, 2.12)	
	40–50 mm	1.46 (1.06, 2.03)	
	≥50 mm	1.38 (1.07, 1.79)	

## Discussion

In this unique and largest series of 580 patients with lung metastases not amenable to surgery and treated with first‐line chemotherapy in EORTC STBSG trials over 30 years, we aimed to find prognostic factors for OS and PFS. So far, outcome of patients with lung metastases only was reported mainly from surgical series. Casson *et al*. in 1992, reported about 58 patients with pulmonary metastases of STS who underwent complete resection of pulmonary metastases. They identified as independent prognostic factors for improved OS: metastasis doubling time of 40 days or greater, unilateral disease on preoperative radiography, three or fewer nodules on preoperative computed tomography, two or less nodules resected, and tumor histology (malignant fibrous histiocytoma better than others). Multivariate analysis identified the number of nodules detected by computed tomography preoperatively as having significant prognostic value.^21^ Later, in 1999, Billingsley *et al*. reported about 719 patients with lung metastases as a single institution experience of the Memorial Sloan‐Kettering Cancer Center. One third of the patients underwent metastasectomy and complete surgical resection of all lung metastasis had the greatest impact on survival. On univariate analysis, patient age 50 years or older and high‐grade tumors were predictors of negative outcome. Again, patients with ≤4 number of lung lesions or unilateral disease had a slightly more favorable survival although this was not statistically significant. For multivariate analysis, surgical resection remained the most significant predictor of post‐metastases survival followed by a disease‐free interval of greater than 12 months and low grade histology of the primary tumor. Predictors of poor outcome were liposarcoma and malignant peripheral nerve sheet tumor histology, and age ≥50 years.^13^

In line with the surgical series, histopathological grade, age and time between initial diagnosis and treatment have been proven to serve as prognostic factors also for the non‐surgically treated patients. The factors PS and involvement of primary site were described for the first time as prognostic in patients with lung metastases only who were treated with first‐line chemotherapy. Whereas the number of metastases or unilaterality were prognostic in the surgical series, the size of the largest lung lesion was prognostic for OS and PFS within this analysis. However, this might be biased by the different way of reporting for chemotherapy trials where tumor load is defined by RECIST criteria, taking into account only measurable lesions (≥10 mm).^22^

STS affects men and women with equal frequency which was also true for this analysis.^23^ However, men were more frequently recorded with lung lesions only as compared to women where lung lesions were more often associated with other metastatic lesions. This is probably related to the gynecologic sarcomas which in a majority of women develop metastases also outside the lungs. Patients with lung lesions only are usually less symptomatic than patients with lesions in liver, bone, or other parts of the body. This lead to an imbalance between the three cohorts with more PS0 patients in the lung only group. This may also explain why for patients with lung metastasis only the delay between initial diagnosis and registration was the longest.

Interestingly, the patients of the cohort with lung lesions only were younger as compared to the others. Whether this is related to different histologies in the three age groups (≤40 years, 40–50 years, and 50–60 years) cannot be clarified as up to 50% of the histologies were grouped as “other” due to the difficulty in verification of histological subtypes over the different study generations, not allowing for further discrimination. However, as only 60% of all cases were analyzed by a review pathologist, the precision of the histologic analysis remains questionable and cannot be compared to more recent studies. We, therefore, also did an analysis on patients where histology was reviewed, but this did not change the conclusions (results not shown).

Extremity STS patients have a higher chance to have achieved complete tumor resection as compared to retroperitoneal or intraperitoneal localizations but they also have a higher risk for pulmonary metastatic disease.^24^ This is in line with the finding that STS patients who had prior complete surgery were more frequently diagnosed with pulmonary lesions only. Radiotherapy was mainly administered in case of extremity STS and was therefore also more often represented in the lung metastases only group. Leiomyosarcoma and “other” STS were the main tumor histologies. Regarding the incidence of sarcoma subtypes especially in metastatic patients undifferentiated pleomorphic sarcoma (UPS) have to be assumed as another large subgroup and aggressive uterine sarcomas in the female population, likely responsible for the higher proportion of lesions outside the lungs in women. The proportion of grade I tumors was below 10% for all patients but significantly higher for patients with metastases outside the lungs. This reflects the low metastatic potential for low grade tumors but may indicate the higher proportion of non‐extremity grade 1 tumors, for example, well‐differentiated liposarcoma in this patient cohort. Patients with lung lesions only originate mainly from extremity primary tumors, which are easier to be completely resected as is the case for other localizations. Therefore, for patients with lung lesions only the primary tumor is more often controlled. This could also explain the better outcome of the lung metastases only group with significantly improved OS and PFS as compared to the other two groups (Figs. 1 *a* and 1 *b*). As the site of tumor metastasis is dependent on the tumor subtype (see Table 1), this result may also reflect the prognosis of the different tumor subtypes with recently describe leiomyosarcoma patients doing better than liposarcoma or undifferentiated pleiomorphic sarcoma.^25^ The involvement of the liver in about 20% of patients and bone in about 10% of patients with metastases outside the lungs is in line with other reports of metastatic STS patients.^26^, ^27^

PS scores are widely used in oncological practice because they correlate with patient survival duration and response to treatment and have proven to serve as independent prognostic factors despite the development of many biomarkers in most cancers.^28^, ^29^ Age did prove as prognostic factor in multivariate analysis but we only have a limited number of about 10% of patients older than 65 years in the EORTC data base treated with first‐line chemotherapy in STS.^30^, ^31^ A longer interval between diagnosis and registration for chemotherapeutic treatment for metastatic disease reflects the clinical course of the disease and is most likely related to lower tumor grade. Histological grade was prognostic for OS but not for PFS reflecting that high grade tumors are more likely to respond to chemotherapy but have also a higher risk to progress thereafter.^28^ With regard to the characteristics of the lung metastases size of the largest lesion was prognostic for OS and PFS. Of note, detection of non‐target lesions in the lungs was associated with the worst outcome. Most likely these patients had pleural involvement which is associated with higher morbidity, compared to intrapulmonary lesions.^29^ Median OS and PFS of patients with pulmonary metastases only was highest for patients treated with doxorubicin plus ifosfamide followed by anthracycline monotherapy, ifosfamide and trabectedin or brostallicin. In the multivariate analysis there was a 31% risk reduction for the combination therapy compared to anthracycline monotherapy with regard to PFS (HR = 0.69, *p* < 0.001). These results are in line with the most recent phase III trial comparing doxorubicin monotherapy with the combination of doxorubicin and ifosfamide (Fig. 2 and 3).^17^

This analysis is restricted by the fact that data encompassing greater than 25 years of sarcoma research were pooled to increase the patient number and clinical significance of the study. With the introduction of modern imaging technology and new treatment options that are more tumor histology driven, prognosis of patients may change in due course. This may also have an impact for analyses such as the current one. Therefore, a stratified approach (by treatment and/or study when feasible in terms of sample size and events in the proposed strata) was adopted. Interestingly, stratification had only a minimal impact on the estimates obtained from the different models when compared to unstratified analyses, suggesting limited presence of heterogeneity. Another limitation of the retrospective nature of our study is the amount of missing data in potentially important prognostic factors such as prior surgery, primary site involved, site of primary, and grade. Treating the missing information as a separate category was a pragmatic solution to avoid the loss of a substantial subgroup of patients in a complete case analysis. Multiple imputation of the missing data was not considered feasible in this analysis, as the proportion of missing data was quite large, and was sometimes of a structural nature (i.e., not being collected as part of the clinical study data).

Despite these limitations, with the largest data set ever investigating the role of localization of metastases in soft tissues sarcoma patients amenable to first‐line chemotherapy, we confirmed general known prognostic factors for OS and PFS but added more detailed information regarding the impact of the status of the primary tumor and the size of the largest pulmonary metastasis.

With this extended report, we aimed to provide significant information on prognostic factors which may help to take optimal decisions in multidisciplinary sarcoma meetings and guide shared decision making with patients confronted with metastases of soft tissue sarcomas.

## Supporting information

Supporting Information
