# Supplementary material for: Prognostic factors for soft tissue sarcoma patients with lung metastases only who are receiving first‐line chemotherapy: An exploratory, retrospective analysis of the European Organization for Research and Treatment of Cancer‐Soft Tissue and Bone Sarcoma Group (EORTC‐STBSG)
Source: Int J Cancer. 2018 Feb 14;142(12):2610–20. doi: 10.1002/ijc.31286 (PMC5947111; doi:10.1002/ijc.31286)
Supplement: Supplementary file 1 — Supporting Information [file IJC-142-2610-s001.docx]

**Appendix**

| **Study versus treatment allocated** | | | | | | |
| --- | --- | --- | --- | --- | --- | --- |
|  | **Treatment** | | | | | **Total (N=2913)** |
|  | **Anthracyclines (N=1118)** | **DOX+IFO (N=948)** | **CYVADIX (N=355)** | **Ifo ALONE (N=335)** | **Other (N=157)** |  |
|  | **N (%)** | **N (%)** | **N (%)** | **N (%)** | **N (%)** | **N (%)** |
| **Protocol** |  |  |  |  |  |  |
| **62012** | 206 (18.4) | 205 (21.6) | 0 (0.0) | 0 (0.0) | 0 (0.0) | 411 (14.1) |
| **62061** | 32 (2.9) | 0 (0.0) | 0 (0.0) | 0 (0.0) | 74 (47.1) | 106 (3.6) |
| **62091** | 38 (3.4) | 0 (0.0) | 0 (0.0) | 0 (0.0) | 83 (52.9) | 121 (4.2) |
| **62761** | 0 (0.0) | 0 (0.0) | 231 (65.1) | 0 (0.0) | 0 (0.0) | 231 (7.9) |
| **62801** | 115 (10.3) | 0 (0.0) | 0 (0.0) | 0 (0.0) | 0 (0.0) | 115 (3.9) |
| **62842** | 0 (0.0) | 149 (15.7) | 0 (0.0) | 0 (0.0) | 0 (0.0) | 149 (5.1) |
| **62851** | 204 (18.2) | 211 (22.3) | 124 (34.9) | 0 (0.0) | 0 (0.0) | 539 (18.5) |
| **62883** | 0 (0.0) | 111 (11.7) | 0 (0.0) | 0 (0.0) | 0 (0.0) | 111 (3.8) |
| **62901** | 319 (28.5) | 0 (0.0) | 0 (0.0) | 0 (0.0) | 0 (0.0) | 319 (11.0) |
| **62903** | 0 (0.0) | 272 (28.7) | 0 (0.0) | 0 (0.0) | 0 (0.0) | 272 (9.3) |
| **62912** | 0 (0.0) | 0 (0.0) | 0 (0.0) | 80 (23.9) | 0 (0.0) | 80 (2.7) |
| **62941** | 36 (3.2) | 0 (0.0) | 0 (0.0) | 0 (0.0) | 0 (0.0) | 36 (1.2) |
| **62953** | 0 (0.0) | 0 (0.0) | 0 (0.0) | 80 (23.9) | 0 (0.0) | 80 (2.7) |
| **62962** | 82 (7.3) | 0 (0.0) | 0 (0.0) | 0 (0.0) | 0 (0.0) | 82 (2.8) |
| **62971** | 86 (7.7) | 0 (0.0) | 0 (0.0) | 175 (52.2) | 0 (0.0) | 261 (9.0) |

Table 1 - Number of patients per study and per allocated treatment

62061: Brostallicin, 62091: Trabectedin

| **Parameter** | **Levels** | **Hazard Ratio (95% CI)** | **P-value** |
| --- | --- | --- | --- |
| Treatment | Anthracyclines | 1.00 | <.001 (df=3) |
|  | DOX+IFO | 0.69 (0.55, 0.87) |  |
|  | Ifo ALONE | 1.21 (0.95, 1.53) |  |
|  | Other | 1.27 (0.89, 1.81) |  |
| Performance status | PS 0 | 1.00 | <.001 (df=2) |
|  | PS 1 | 1.28 (1.06, 1.55) |  |
|  | PS 2+ | 3.13 (1.63, 6.02) |  |
| Age | <= 40 yrs | 1.00 | 0.039 (df=3) |
|  | 40-50 yrs | 0.91 (0.70, 1.18) |  |
|  | 50-60 yrs | 1.11 (0.86, 1.42) |  |
|  | > 60 yrs | 1.37 (1.03, 1.82) |  |
| Time between initial diagnosis | <=6 mon | 1.00 | <.001 (df=3) |
|  | 1-2 yrs | 0.91 (0.71, 1.18) |  |
|  | 6-12 mon | 1.27 (0.97, 1.67) |  |
|  | > 2yrs | 0.62 (0.49, 0.79) |  |
| Size of largest lung lesion | <20 mm | 1.00 | 0.011 (df=4) |
|  | 20-30 mm | 1.24 (0.96, 1.61) |  |
|  | 30-40 mm | 1.60 (1.17, 2.18) |  |
|  | 40-50 mm | 1.53 (1.10, 2.12) |  |
|  | >=50 mm | 1.42 (1.09, 1.83) |  |

Appendix Table 2 – Histological subtypes

|  | **Lesions** | | | **Total (N=1451)** |
| --- | --- | --- | --- | --- |
|  | **Lung lesions only (N=502)** | **Other lesions only (N=466)** | **Both (N=483)** |  |
|  | **N (%)** | **N (%)** | **N (%)** | **N (%)** |
| **Site of primary tumor** |  |  |  |  |
| **Head & Neck** | 6 (1.2) | 8 (1.7) | 10 (2.1) | 24 (1.7) |
| **Skin** | 0 (0.0) | 2 (0.4) | 5 (1.0) | 7 (0.5) |
| **Trunk** | 21 (4.2) | 6 (1.3) | 23 (4.8) | 50 (3.4) |
| **Thoracic** | 21 (4.2) | 11 (2.4) | 19 (3.9) | 51 (3.5) |
| **Abdominal** | 25 (5.0) | 94 (20.2) | 46 (9.5) | 165 (11.4) |
| **Lower extremity** | 121 (24.1) | 43 (9.2) | 96 (19.9) | 260 (17.9) |
| **Upper extremity** | 30 (6.0) | 11 (2.4) | 21 (4.3) | 62 (4.3) |
| **GU** | 1 (0.2) | 4 (0.9) | 3 (0.6) | 8 (0.6) |
| **GI** | 4 (0.8) | 43 (9.2) | 15 (3.1) | 62 (4.3) |
| **Gynaecological** | 117 (23.3) | 73 (15.7) | 118 (24.4) | 308 (21.2) |
| **Breast** | 7 (1.4) | 10 (2.1) | 10 (2.1) | 27 (1.9) |
| **Other viscera** | 0 (0.0) | 7 (1.5) | 3 (0.6) | 10 (0.7) |
| **Other** | 3 (0.6) | 5 (1.1) | 4 (0.8) | 12 (0.8) |
| ***Missing*** | 146 (29.1) | 149 (32.0) | 110 (22.8) | 405 (27.9) |

Appendix Table 3 – Site of origin of the tumors in female patients only

| **Covariates** |  | **Patients (N)** | **Observed Events (O)** | **Median (95% CI) (Years)** | **% at 1 Year(s) (95% CI)** | **Hazard Ratio (95% CI)** | **P-Value (Score test)** |
| --- | --- | --- | --- | --- | --- | --- | --- |
| **Treatment** | Anthracyclines | 215 | 171 | 1.04 (0.87, 1.28) | 51.7 (44.6, 58.4) | 1.00 | 0.041 (df=3) |
|  | DOX+IFO | 181 | 162 | 1.19 (1.05, 1.38) | 60.2 (52.6, 66.9) | 0.85 (0.68, 1.05) |  |
|  | Ifo ALONE | 138 | 116 | 0.90 (0.77, 1.05) | 44.6 (35.9, 52.9) | 1.18 (0.93, 1.49) |  |
|  | Other | 46 | 18 | 1.57 (0.73, N) | 62.0 (45.2, 75.1) | 0.78 (0.48, 1.27) |  |

| **Covariates (stratified by treatment)** | | **Patients (N)** | **Observed Events (O)** | **Median (95% CI) (Years)** | **% at 1 Year(s) (95% CI)** | **Hazard Ratio (95% CI)** | **P-Value (Score test)** |
| --- | --- | --- | --- | --- | --- | --- | --- |
| **Gender** | Male | 307 | 247 | 1.05 (0.92, 1.20) | 52.7 (46.8, 58.3) | 1.00 | 0.838 |
|  | Female | 273 | 220 | 1.12 (0.98, 1.28) | 53.7 (47.4, 59.6) | 0.98 (0.82, 1.18) |  |
| **Performance status** | PS 0 | 314 | 235 | 1.36 (1.22, 1.57) | 66.1 (60.4, 71.2) | 1.00 | <0.001 (df=2) |
|  | PS 1 | 253 | 219 | 0.82 (0.74, 0.91) | 39.4 (33.2, 45.6) | 1.89 (1.56, 2.28) |  |
|  | PS 2+ | 11 | 11 | 0.29 (0.06, 0.80) | 9.1 (0.5, 33.3) | 5.22 (2.80, 9.73) |  |
| **Primary site involved** | No | 313 | 242 | 1.13 (1.02, 1.29) | 56.6 (50.7, 62.1) | 1.00 | 0.134 (df=2) |
|  | Yes | 174 | 147 | 0.98 (0.83, 1.16) | 47.9 (40.1, 55.2) | 1.20 (0.97, 1.47) |  |
|  | *Missing* | *93* | *78* | *1.02 (0.87, 1.45)* | *52.1 (41.2, 61.8)* | *1.22 (0.93, 1.60)* |  |
| **Histology** | Leiomyosarcoma | 154 | 123 | 1.03 (0.90, 1.28) | 51.7 (43.2, 59.6) | 1.00 | 0.386 (df=3) |
|  | Synovial sarcoma | 107 | 90 | 1.28 (1.18, 1.62) | 70.4 (60.6, 78.2) | 0.80 (0.61, 1.06) |  |
|  | Liposarcoma | 36 | 29 | 1.29 (0.83, 1.65) | 57.6 (38.9, 72.4) | 1.04 (0.69, 1.56) |  |
|  | Other | 273 | 217 | 0.92 (0.82, 1.05) | 46.3 (40.1, 52.3) | 0.96 (0.77, 1.20) |  |
| **Grade** | Grade I | 50 | 36 | 1.51 (1.11, 2.66) | 67.3 (52.2, 78.5) | 1.00 | <0.001 (df=3) |
|  | Grade II | 186 | 152 | 1.24 (1.07, 1.45) | 59.8 (52.2, 66.5) | 1.40 (0.97, 2.02) |  |
|  | Grade III | 210 | 187 | 0.98 (0.85, 1.05) | 46.3 (39.4, 52.9) | 2.05 (1.43, 2.95) |  |
|  | *Missing* | *134* | *92* | *1.02 (0.74, 1.29)* | *50.2 (40.6, 59.1)* | *1.95 (1.31, 2.88)* |  |
| **Site of primary** | Other | 213 | 172 | 0.92 (0.82, 1.10) | 45.7 (38.7, 52.4) | 1.00 | 0.067 (df=2) |
|  | Extr | 242 | 192 | 1.19 (1.04, 1.36) | 59.0 (52.3, 65.0) | 0.80 (0.65, 0.99) |  |
|  | *Missing* | *125* | *103* | *1.12 (0.85, 1.24)* | *54.6 (45.1, 63.2)* | *1.01 (0.78, 1.29)* |  |
| **Number of measured** | 0 | 45 | 35 | 1.19 (0.91, 1.51) | 56.1 (40.2, 69.4) | 1.00 | 0.133 (df=3) |
| **lung lesions** | 1 | 194 | 149 | 1.20 (0.98, 1.41) | 56.9 (49.4, 63.8) | 0.99 (0.68, 1.43) |  |
|  | 2 | 166 | 121 | 1.02 (0.87, 1.20) | 50.4 (42.1, 58.2) | 1.22 (0.83, 1.79) |  |
|  | 3+ | 175 | 162 | 1.04 (0.85, 1.15) | 51.2 (43.5, 58.3) | 1.27 (0.87, 1.86) |  |
| **Age** | <= 40 yrs | 145 | 127 | 1.18 (0.99, 1.45) | 58.4 (49.9, 66.1) | 1.00 | 0.115 (df=3) |
|  | 40-50 yrs | 140 | 112 | 1.10 (0.89, 1.29) | 52.5 (43.7, 60.6) | 1.00 (0.77, 1.29) |  |
|  | 50-60 yrs | 174 | 135 | 1.10 (0.94, 1.29) | 53.2 (45.2, 60.5) | 1.11 (0.87, 1.43) |  |
|  | > 60 yrs | 121 | 93 | 0.98 (0.73, 1.16) | 47.8 (38.2, 56.8) | 1.36 (1.03, 1.79) |  |
| **Time between initial** | <=6 mon | 242 | 203 | 0.87 (0.80, 1.04) | 45.4 (38.8, 51.7) | 1.00 | <0.001 (df=3) |
| **diagnosis and start** | 6-12 mon | 78 | 66 | 0.92 (0.73, 1.16) | 46.9 (35.2, 57.7) | 1.00 (0.75, 1.32) |  |
| **of treatment** | 1-2 yrs | 99 | 83 | 1.08 (0.89, 1.36) | 55.6 (44.9, 65.1) | 0.88 (0.68, 1.14) |  |
|  | > 2yrs | 132 | 88 | 1.60 (1.29, 1.98) | 68.4 (59.3, 75.8) | 0.52 (0.41, 0.68) |  |
| **Size of largest lung** | <20 mm | 158 | 115 | 1.52 (1.25, 1.64) | 67.3 (59.1, 74.3) | 1.00 | <0.001 (df=4) |
| **lesion** | 20-30 mm | 115 | 92 | 1.20 (1.00, 1.44) | 60.1 (50.3, 68.6) | 1.23 (0.93, 1.63) |  |
|  | 30-40 mm | 63 | 56 | 0.87 (0.69, 1.13) | 43.6 (30.9, 55.7) | 1.77 (1.28, 2.45) |  |
|  | 40-50 mm | 61 | 50 | 1.02 (0.60, 1.20) | 50.9 (37.2, 63.0) | 1.66 (1.18, 2.33) |  |
|  | >=50 mm | 138 | 119 | 0.81 (0.71, 0.87) | 36.2 (28.1, 44.4) | 1.89 (1.46, 2.46) |  |

Appendix Table 4 – Univariate analysis of prognostic factors for overall survival for patients with lung metastasis only.

| **Covariates** |  | **Patients (N)** | **Observed Events (O)** | **Median (95% CI) (Years)** | **% at 0.5 Year(s) (95% CI)** | **Hazard Ratio (95% CI)** | **P-Value (Score test)** |
| --- | --- | --- | --- | --- | --- | --- | --- |
| **Treatment** | Anthracyclines | 215 | 207 | 0.37 (0.28, 0.44) | 36.4 (30.0, 42.9) | 1.00 | <0.001 (df=3) |
|  | DOX+IFO | 181 | 175 | 0.57 (0.53, 0.66) | 62.2 (54.7, 68.8) | 0.61 (0.50, 0.75) |  |
|  | Ifo ALONE | 138 | 135 | 0.29 (0.23, 0.36) | 31.0 (23.5, 38.8) | 1.15 (0.93, 1.43) |  |
|  | Other | 46 | 46 | 0.20 (0.12, 0.25) | 28.3 (16.2, 41.6) | 1.24 (0.90, 1.70) |  |

| **Covariates (stratified by treatment)** |  | **Patients (N)** | **Observed Events (O)** | **Median (95% CI) (Years)** | **% at 0.5 Year(s) (95% CI)** | **Hazard Ratio (95% CI)** | **P-Value (Score test)** |
| --- | --- | --- | --- | --- | --- | --- | --- |
| **Gender** | Male | 307 | 295 | 0.38 (0.31, 0.44) | 40.0 (34.5, 45.5) | 1.00 | 0.528 |
|  | Female | 273 | 268 | 0.46 (0.35, 0.51) | 45.3 (39.3, 51.1) | 0.95 (0.80, 1.12) |  |
| **Performance status** | PS 0 | 314 | 304 | 0.50 (0.44, 0.54) | 50.2 (44.6, 55.6) | 1.00 | <0.001 (df=2) |
|  | PS 1 | 253 | 246 | 0.33 (0.24, 0.41) | 34.7 (28.8, 40.6) | 1.42 (1.19, 1.69) |  |
|  | PS 2+ | 11 | 11 | 0.11 (0.04, 0.17) | 9.1 (0.5, 33.3) | 3.28 (1.77, 6.06) |  |
| **Primary site involved** | No | 313 | 304 | 0.48 (0.40, 0.51) | 45.2 (39.6, 50.6) | 1.00 | 0.253 (df=2) |
|  | Yes | 174 | 171 | 0.30 (0.23, 0.39) | 36.5 (29.4, 43.7) | 1.17 (0.97, 1.41) |  |
|  | *Missing* | *93* | *88* | *0.44 (0.27, 0.57)* | *44.8 (34.5, 54.6)* | *1.02 (0.79, 1.30)* |  |
| **Histology** | Leiomyosarcoma | 154 | 152 | 0.44 (0.31, 0.51) | 43.8 (35.8, 51.5) | 1.00 | 0.140 (df=3) |
|  | Synovial sarcoma | 107 | 105 | 0.56 (0.50, 0.62) | 61.5 (51.6, 70.0) | 0.76 (0.59, 0.97) |  |
|  | Liposarcoma | 36 | 35 | 0.41 (0.23, 0.57) | 41.7 (25.6, 57.0) | 0.86 (0.59, 1.25) |  |
|  | Other | 273 | 261 | 0.29 (0.25, 0.37) | 33.4 (27.9, 39.1) | 0.96 (0.78, 1.17) |  |
| **Grade** | Grade I | 50 | 49 | 0.41 (0.29, 0.52) | 40.0 (26.5, 53.1) | 1.00 | 0.182 (df=3) |
|  | Grade II | 186 | 182 | 0.48 (0.38, 0.54) | 48.8 (41.5, 55.8) | 1.10 (0.80, 1.51) |  |
|  | Grade III | 210 | 205 | 0.39 (0.28, 0.49) | 41.9 (35.2, 48.5) | 1.32 (0.96, 1.81) |  |
|  | *Missing* | *134* | *127* | *0.36 (0.27, 0.47)* | *35.4 (27.3, 43.6)* | *1.23 (0.87, 1.72)* |  |
| **Site of primary** | Other | 213 | 207 | 0.37 (0.29, 0.41) | 36.3 (29.8, 42.7) | 1.00 | 0.491 (df=2) |
|  | Extr | 242 | 236 | 0.47 (0.36, 0.52) | 46.6 (40.2, 52.8) | 0.90 (0.75, 1.09) |  |
|  | *Missing* | *125* | *120* | *0.46 (0.35, 0.51)* | *45.1 (36.2, 53.6)* | *1.00 (0.79, 1.26)* |  |
| **Number of measured** | 0 | 45 | 44 | 0.24 (0.17, 0.44) | 31.1 (18.4, 44.7) | 1.00 | 0.049 (df=3) |
| **lung lesions** | 1 | 194 | 187 | 0.46 (0.35, 0.52) | 45.7 (38.5, 52.5) | 0.75 (0.54, 1.05) |  |
|  | 2 | 166 | 161 | 0.31 (0.24, 0.46) | 35.5 (28.2, 42.8) | 0.97 (0.68, 1.37) |  |
|  | 3+ | 175 | 171 | 0.48 (0.38, 0.53) | 48.6 (41.0, 55.7) | 0.96 (0.68, 1.36) |  |
| **Age** | <= 40 yrs | 145 | 143 | 0.49 (0.41, 0.54) | 49.0 (40.6, 56.8) | 1.00 | 0.246 (df=3) |
|  | 40-50 yrs | 140 | 132 | 0.44 (0.35, 0.56) | 45.0 (36.5, 53.1) | 1.01 (0.80, 1.29) |  |
|  | 50-60 yrs | 174 | 170 | 0.41 (0.30, 0.51) | 43.1 (35.7, 50.3) | 1.15 (0.91, 1.44) |  |
|  | > 60 yrs | 121 | 118 | 0.25 (0.19, 0.34) | 31.0 (23.0, 39.4) | 1.26 (0.97, 1.63) |  |
| **Time between initial** | <=6 mon | 242 | 234 | 0.34 (0.26, 0.41) | 36.3 (30.3, 42.4) | 1.00 | <0.001 (df=3) |
| **diagnosis and start** | 6-12 mon | 78 | 78 | 0.30 (0.19, 0.41) | 30.8 (20.9, 41.1) | 1.10 (0.85, 1.42) |  |
| **of treatment** | 1-2 yrs | 99 | 97 | 0.44 (0.30, 0.53) | 45.5 (35.5, 54.9) | 0.95 (0.75, 1.21) |  |
|  | > 2yrs | 132 | 125 | 0.56 (0.47, 0.66) | 55.9 (46.9, 63.9) | 0.65 (0.52, 0.81) |  |
| **Size of largest lung** | <20 mm | 158 | 152 | 0.59 (0.49, 0.67) | 57.3 (49.2, 64.6) | 1.00 | 0.004 (df=4) |
| **lesion** | 20-30 mm | 115 | 112 | 0.40 (0.29, 0.49) | 39.6 (30.6, 48.4) | 1.39 (1.08, 1.79) |  |
|  | 30-40 mm | 63 | 63 | 0.46 (0.29, 0.56) | 42.9 (30.5, 54.6) | 1.36 (1.01, 1.84) |  |
|  | 40-50 mm | 61 | 61 | 0.26 (0.19, 0.39) | 27.9 (17.3, 39.4) | 1.71 (1.26, 2.32) |  |
|  | >=50 mm | 138 | 131 | 0.35 (0.26, 0.46) | 38.1 (30.0, 46.2) | 1.37 (1.07, 1.74) |  |

Appendix Table 5 – Univariate analysis of prognostic factors for progression free survival for patients with lung metastasis only.

| **Parameter** | **Levels** | **Hazard Ratio (95% CI)** | **P-value** |
| --- | --- | --- | --- |
| Performance status | PS 0 | 1.00 | <.001 (df=2) |
|  | PS 1 | 1.57 (1.21, 2.03) |  |
|  | PS 2+ | 6.44 (2.81, 14.77) |  |
| Histopathological grade | Grade I | 1.00 | 0.018 (df=3) |
|  | Grade II | 1.35 (0.85, 2.15) |  |
|  | Grade III | 1.83 (1.16, 2.90) |  |
|  | Missing | 2.85 (0.62, 13.11) |  |
| Age | <= 40 yrs | 1.00 | 0.048 (df=3) |
|  | 40-50 yrs | 0.79 (0.56, 1.12) |  |
|  | 50-60 yrs | 1.00 (0.71, 1.40) |  |
|  | > 60 yrs | 1.37 (0.93, 2.02) |  |
| Time between initial diagnosis | <=6 mon | 1.00 | <.001 (df=3) |
|  | 1-2 yrs | 0.82 (0.58, 1.16) |  |
|  | 6-12 mon | 1.12 (0.79, 1.58) |  |
|  | > 2yrs | 0.52 (0.37, 0.73) |  |
| Size of largest lung lesion | <20 mm | 1.00 | <.001 (df=4) |
|  | 20-30 mm | 1.41 (0.98, 2.02) |  |
|  | 30-40 mm | 2.34 (1.52, 3.58) |  |
|  | 40-50 mm | 1.36 (0.84, 2.21) |  |
|  | >=50 mm | 2.38 (1.67, 3.39) |  |

Appendix Table 6 – Overall survival - centrally reviewed cases only (N = 331)

| **Parameter** | **Levels** | **Hazard Ratio (95% CI)** | **P-value** |
| --- | --- | --- | --- |
| Performance status | PS 2+ | 1.00 | <.001 (df=2) |
|  | PS 0 | 0.26 (0.11, 0.59) |  |
|  | PS 1 | 0.37 (0.16, 0.84) |  |
| Site of primary tumor | Other | 1.00 | 0.036 (df=2) |
|  | Extr | 0.99 (0.77, 1.28) |  |
|  | Missing | 1.48 (1.06, 2.06) |  |
| Time between initial diagnosis | > 2yrs | 1.00 | 0.003 (df=3) |
|  | 1-2 yrs | 1.27 (0.89, 1.81) |  |
|  | 6-12 mon | 1.80 (1.25, 2.60) |  |
|  | <=6 mon | 1.63 (1.21, 2.19) |  |
| Size of largest lung lesion | >=50 mm | 1.00 | 0.040 (df=4) |
|  | 20-30 mm | 1.01 (0.73, 1.40) |  |
|  | 30-40 mm | 1.13 (0.77, 1.65) |  |
|  | 40-50 mm | 0.95 (0.62, 1.46) |  |
|  | <20 mm | 0.67 (0.49, 0.93) |  |

Appendix Table 7 - Progression free survival - centrally reviewed cases only (N = 331)
